# Supplementary material for: Netrin-1 disrupt high-fat-diet-induced adipogenesis via the PPARγ and Wnt/β-catenin signaling pathways
Source: Commun Biol. 2026 Feb 21;9:471. doi: 10.1038/s42003-026-09749-x (PMC13035891; doi:10.1038/s42003-026-09749-x)

Supplemental Figures

Supplementary Figure 1. Adipose-specific deletion of Netrin-1 in C57BL6 mice.

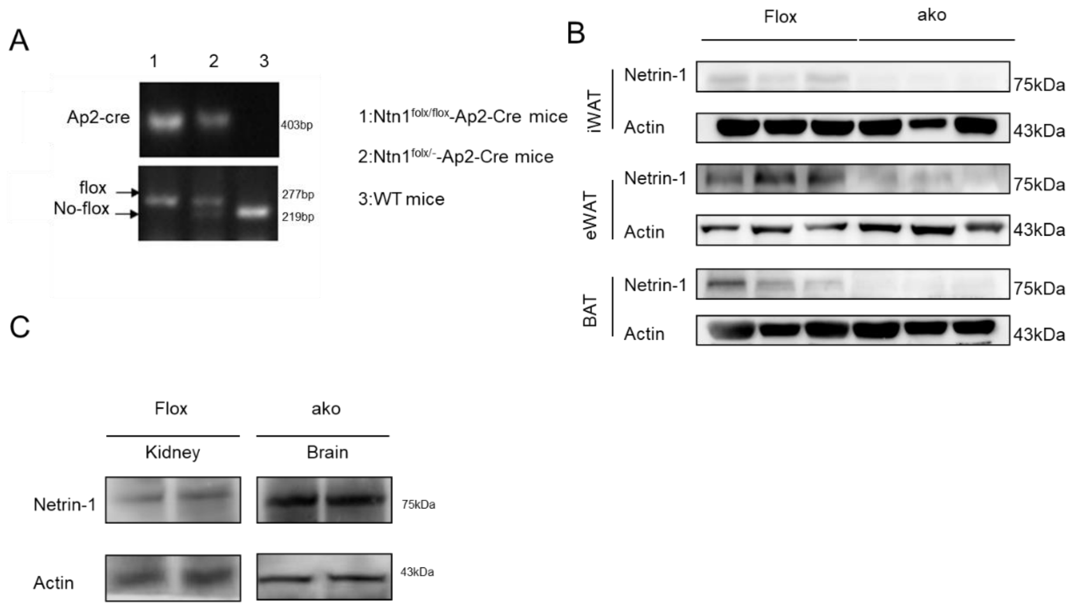

Fig. S1 (A) Genotyping of the Ntn1^{AKO} heterozygous and Flox mice. (B) The expression of Netrin-1 in the iWATs, eWATs and BATs of the Ntn1^{AKO} mice. (C) Expression of Netrin-1 in other tissues from the Ntn1^{AKO} mice.

Supplementary Figure 2. Improved insulin signalling in mature adipocytes that lack Netrin-1 expression.

A

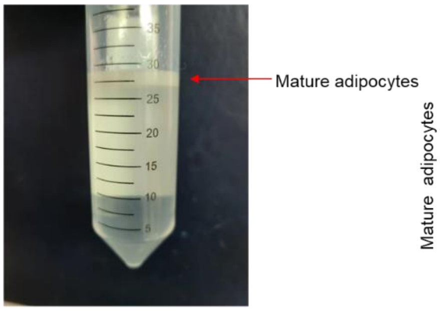

B

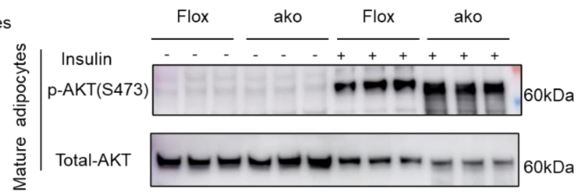

Fig. S2 (A) Mature adipocytes isolated from the WATs of Ntn1^{AKO} mice. (B) Western blot analysis of p-AKT (Ser473) and total AKT expression from insulin-exposed primary adipocytes obtained from the Ntn1^{AKO} mice.

Supplementary Figure 3. No differences were observed in body composition between the Ntn1^{AKO} and Flox mice that were maintained on chow diet.

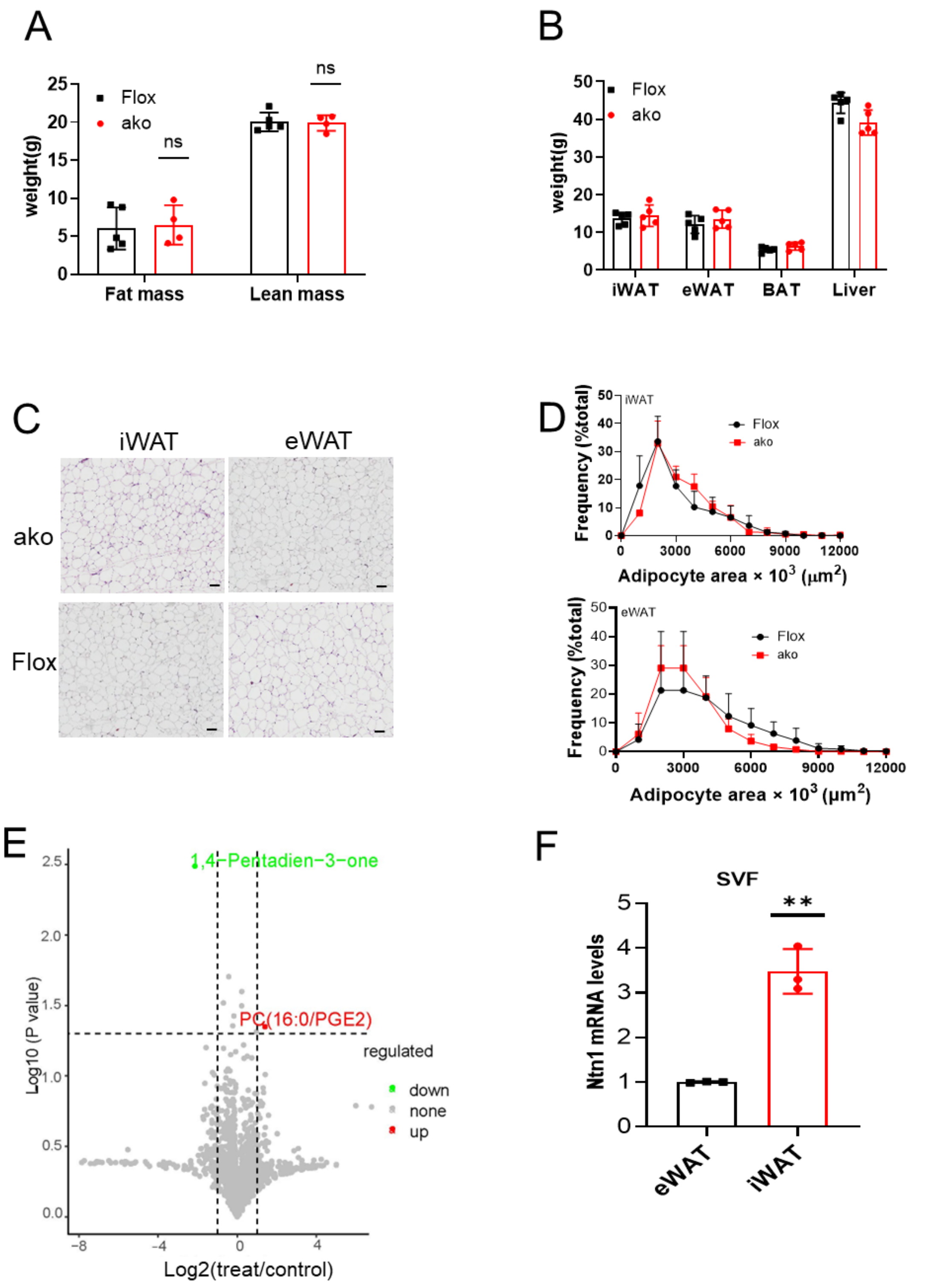

Fig. S3 (A)Average weights of the fat mass and lean mass in the chow-fed Ntn-1^{AKO} and

Flox mice, n=4-5 per group. (B) Tissue index of the iWAT, eWAT, BAT and liver (average weights of different tissues normalized to body weight) from the high-fat-fed Flox and $Ntn1^{AKO}$ mice, n=5 per group. (C) Representative H&E staining images of iWAT and eWAT from Flox and $Ntn1^{AKO}$ mice fed on HFD for eight weeks. (D) Adipocyte area of iWAT and eWAT from Flox and $Ntn1^{AKO}$ mice fed on HFD for eight weeks. (E) Changes in metabolites following Netrin-1 knockout in adipose tissue, red upregulated and green downregulated. (F) The mRNA levels of Netrin-1 in the SVFs isolated from eWAT and iWAT of normal chow-fed Flox mice. Data are shown as mean ± s.e.m. *P < 0.05, **P<0.01 ***P<0.001 over Control as analysed by Student's t-test, ns means not significant. Scale bar=100 μm.

Supplementary Figure 4. Improved WAT fibrosis in the high-fat- fed Ntn1 ^{AKO} mice.

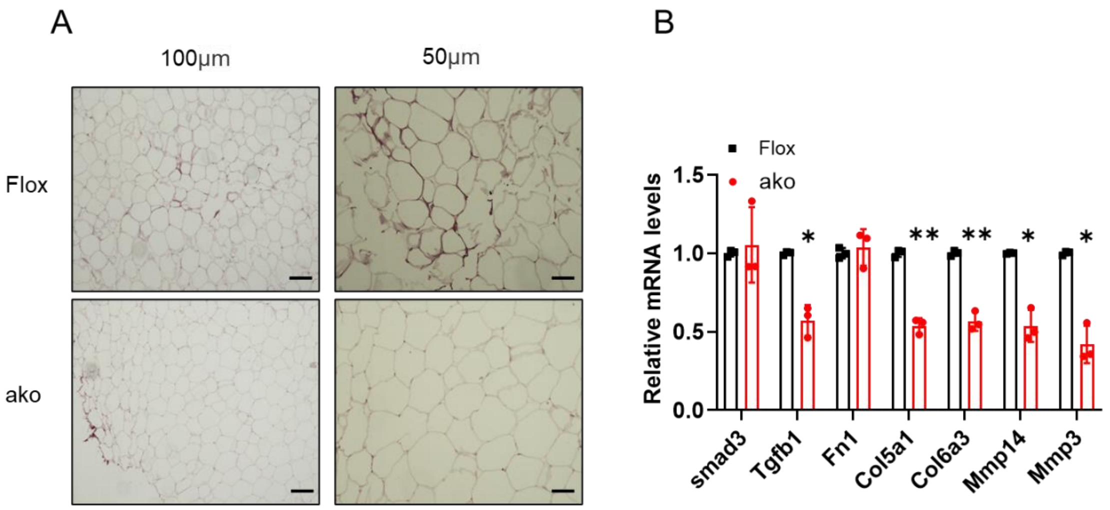

Fig.S4 (A) Representative images of picrosirius red staining of the WATs extracted from the high-fat-fed Ntn1^{AKO} and Flox mice. Scale bar=100 μm (left); Scale bar=50 μm (right). (B) Results of qPCR analysis of genes that are involved in extracellular matrix composition synthesis using WATs extracted from the high-fat-fed Ntn-1^{AKO} and Flox mice, n=3 per group. Data are shown as mean ± s.e.m. *$P < 0.05$, **$P<0.01$ over Control as analysed by Student's t-test.

Supplementary Figure 5. Expression of Netrin-1 following in vivo AAV-Ntn1 administration.

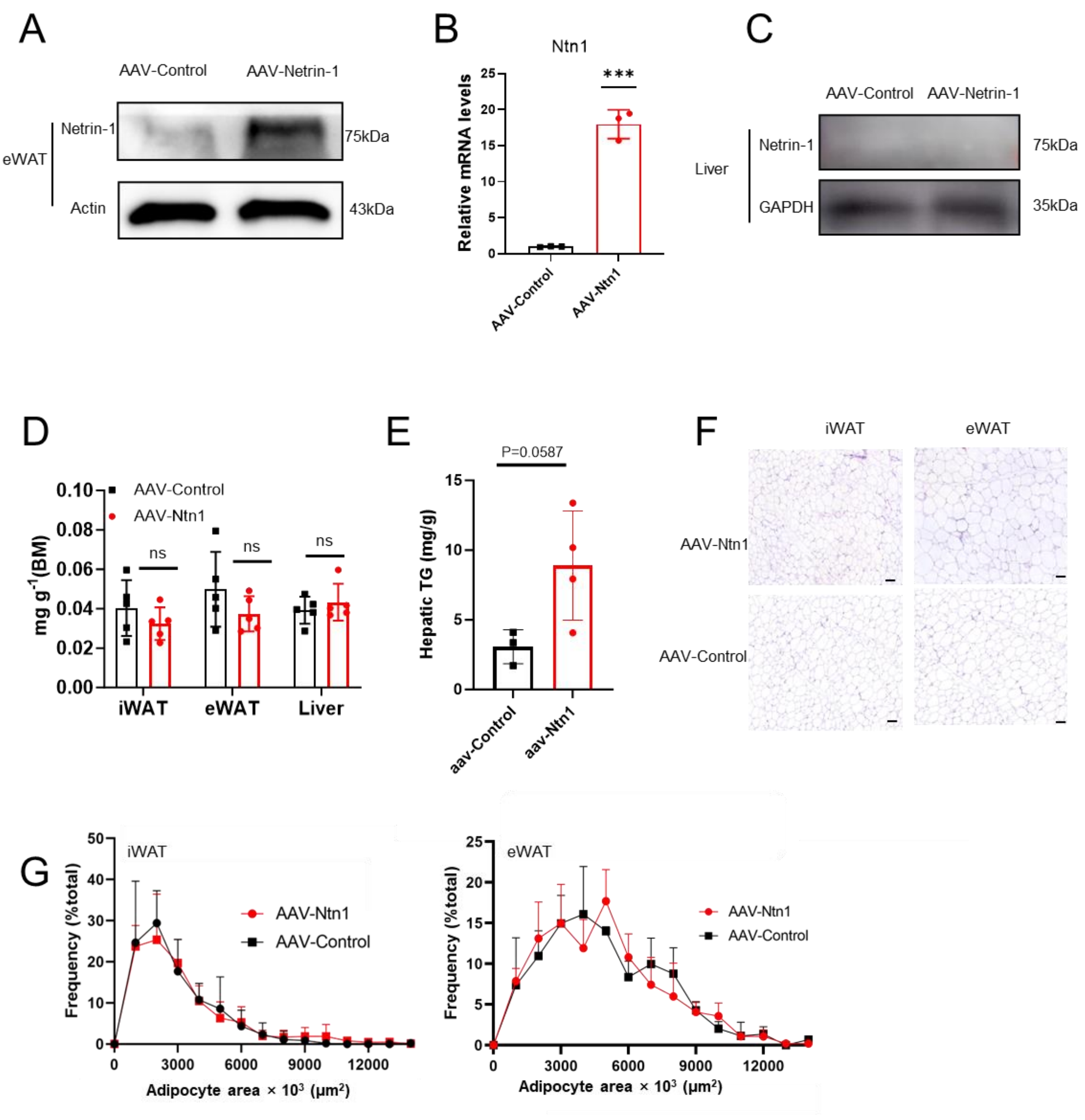

Fig. S5 (A-B) Upregulation of Netrin-1 was observed in the eWATs extracted from the AAV-Ntn1-transfected mice both on protein (a) and mRNA (B) levels. (C) Expression of Netrin-1 protein level was unchanged in liver following in vivo delivery of AAV-Ntn1. (D) Tissue index (weights of specific tissue normalized to body weight) of iWAT, eWAT, BAT and liver from AAV-Control and AAV-Ntn1 mice fed on HFD for eight weeks, n=5 per group. (E) Triglyceride concentration of liver from AAV-Ntn1 and

AAV-Control mice fed on HFD (F) Representative images of H&E staining analysis of the iWATs and eWATs extracted from the high-fat-fed AAV-Ntn1 and AAV-Control mice. (G) Adipocyte area of iWAT and eWAT after AAV-Netrin-1 and AAV-Control delivered. Data are shown as mean ± s.e.m. ***P<0.001 over Control as analysed by Student's t-test.

Supplementary Figure 6. WATs-specific Netrin-1 deficiency resulted in altered expression of genes involved in multiple energy metabolism pathways and the expression of Netrin-1 was downregulated by adipogenesis.

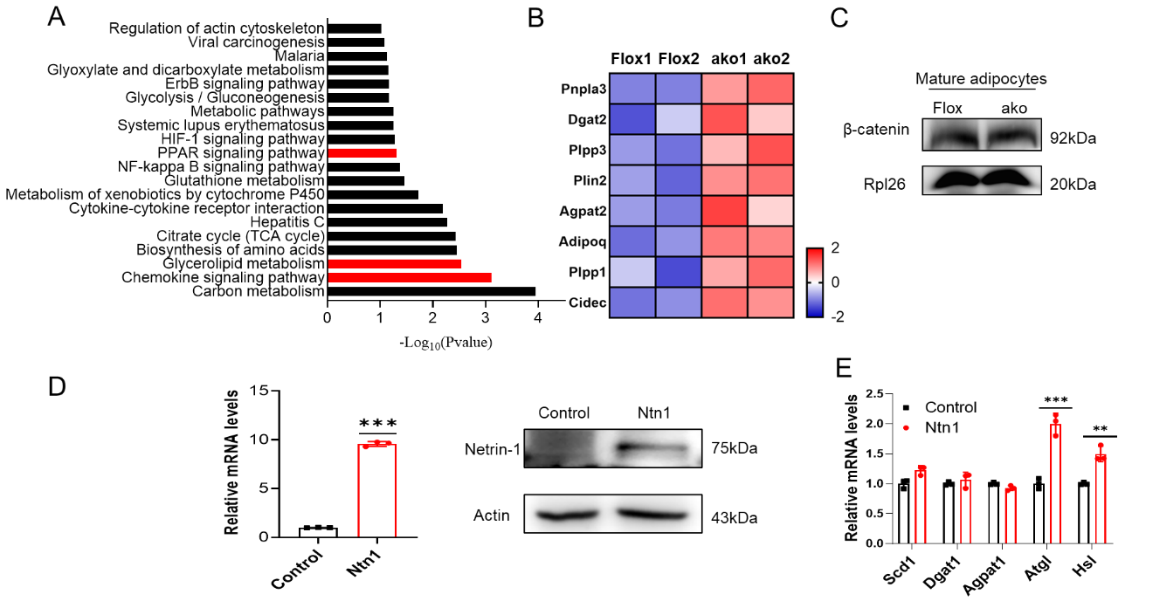

Fig. S6 (A)The KEGG analysis of WATs extracted from the high-fat-fed Ntn1^{AKO} mice. Pathways involved in energy metabolism were enriched and highlighted. (B) Genes involved in glycerolipid metabolism were upregulated as a result of WAT-specific Netrin-1 deficiency. (C) The protein level of β-catenin in the mature adipocytes isolated from the high-fat-fed Flox and Ntn1AKO mice. (D) The mRNA and protein levels of Netrin-1 from 3T3-L1-derived mature adipocytes that overexpress Netrin-1. (E) The mRNA levels of genes that are involved in triglyceride synthesis and lipogenesis in Netrin-1-overexpressing 3T3-L1-derived mature adipocytes. Data are shown as mean ± s.e.m. **P<0.01 ***P<0.001 over Control as analysed by Student's t-test.

Supplementary Figure 7. Netrin-1 inhibit adipogenesis via the WNT signaling pathway

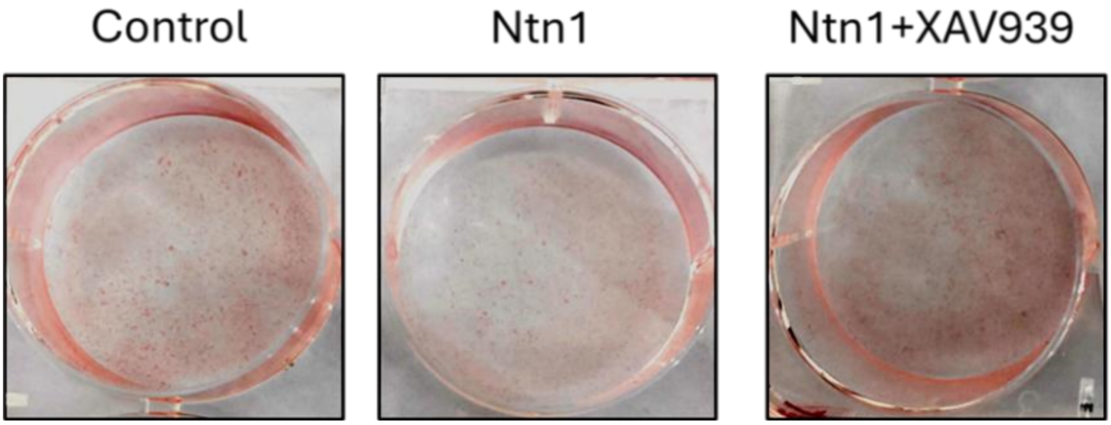

Fig. S7. Representative Oil Red O staining images of SVF cells on day 6 of adipogenic differentiation in Control, Netrin-1 overexpression (Ntn1), and Ntn1+XAV939 groups

Supplementary Figure 8

Western blot quantification results

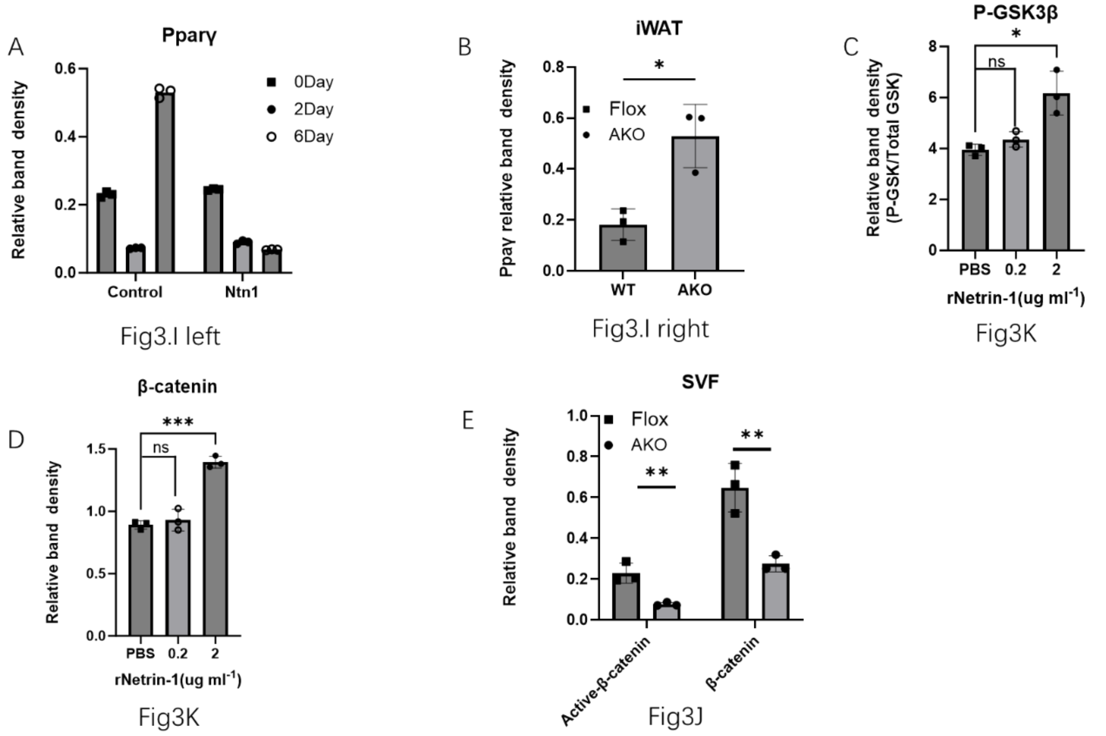

Fig. S8. Quantitative analysis (densitometry) of corresponding western blotting data. (A-B) Quantitative analysis of PPARγ expression levels as presented in Figure 3I left panel (A) and Figure 3 I right panel (B). (C) Quantitative analysis of phospho-GSK3β as presented in Figure 3K. (D-E) Quantitative analysis of β-catenin as presented in Figure 3K (D) and Figure 3J (E). (F) Qantitative analysis of Netrin-1 expression as presented in Figure 4E.

Supplementary Table 1. Primer sequences used for real-time quantitative PCR and ChIP analysis.

Gene symbol	Forward primer	Reverse primer
Ntn1	CAGCCTGATCCTTGCTCGG	GCGGGTTATTGAGGTCGGTG
Pparγ	TCGCTGATGCACTGCCTATG	GAGAGGTCCACAGAGCTGATT
Smad3	AGGGGCTCCCTCACGTTATC	CATGGCCCGTAATTCATGGTG
Tgfb1	CCACCTGCAAGACCATCGAC	CTGGCGAGCCTTAGTTTGGAC
Fn1	ATGTGGACCCCTCCTGATAT	GCCCAGTGATTTCAGCAAAGG
Col5a1	CTTCGCCGCTACTCCTGTTC	CCCTGAGGGCAAATTGTGAAAA
Col6a3	GCTGCGGAATCACTTTGTGC	CACCTTGACACCTTTCTGGGT
Mmp3	TGATGAACGATGGACAGAGG	GAGAGATGGAAACGGGACAA
Mmp14	CAGTATGGCTACCTACCTCCAG	GCCTTGCCTGTCACTTGTAAA
Plin1	CTGTGTGCAATGCCTATGAGA	CTGGAGGGTATTGAAGAGCCG
C/ebpa	CAAGAACAGCAACGAGTACCG	GTCACTGGTCAGCTCCAGCAC
Ucp1	AGGCTTCCAGTACCATTAGGT	CTGAGTGAGGCAAAGCTGATTT
ChIP-primers<br>Ntn1	AGGACACCCCAGGAGACT<br>TTGGCTTTAACTTTCTTCTTCC	GTGTGCTTTTGCCACTTCA<br>CACACACACACACACACTCACG

Supplementary Figure 9: Uncropped blots

Uncropped blots

Fig2 H

Right pannel

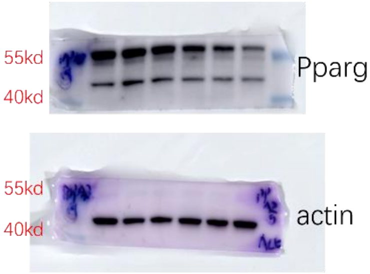

Left pannel

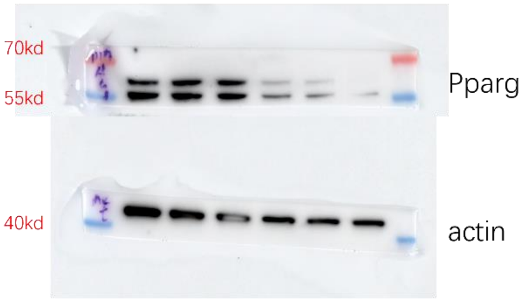

Fig2 I

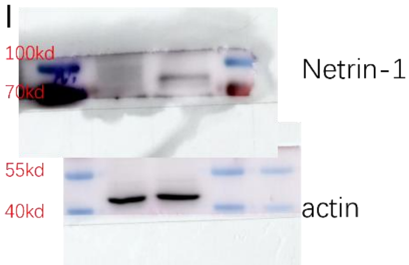

Uncropped blots

Fig4 E

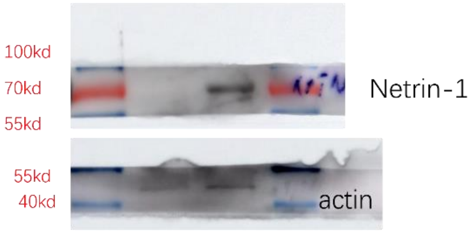

Fig5D

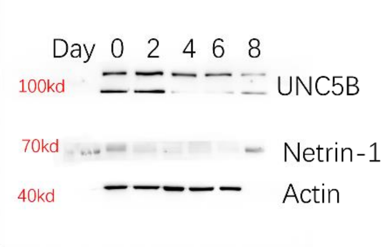

Fig5 E

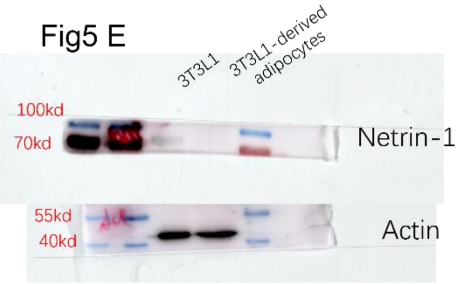

Fig5 F

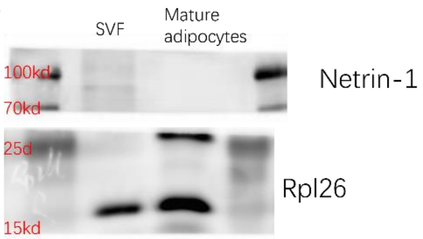

Uncropped blots

Fig5G

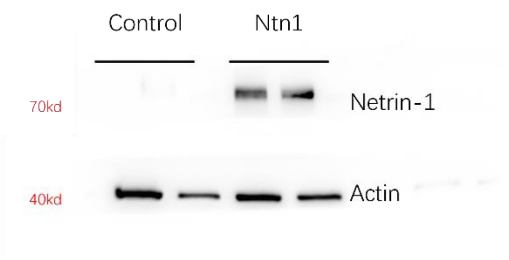

Uncropped blots

Fig5 i

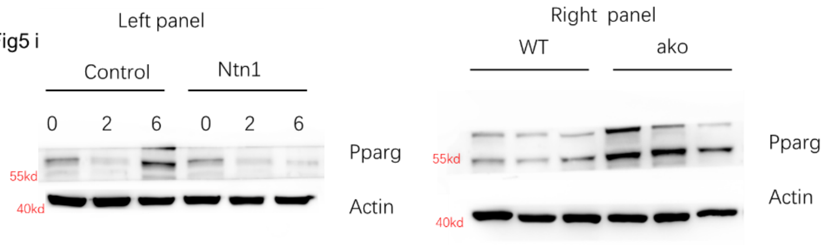

Fig5 K

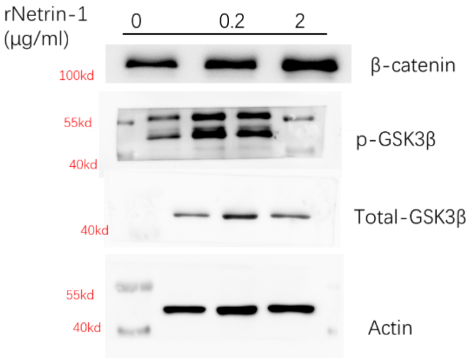

Fig5 L

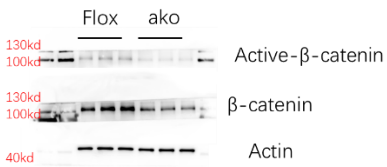

Uncropped blots

Fig6 A

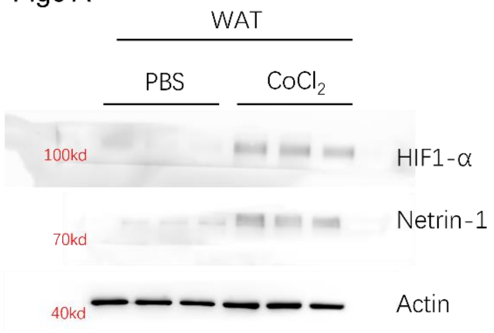

Fig6 C

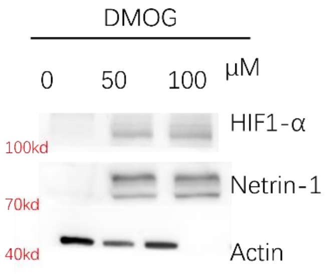

Supplementary Figure 9
Uncropped blots

Fig6 E

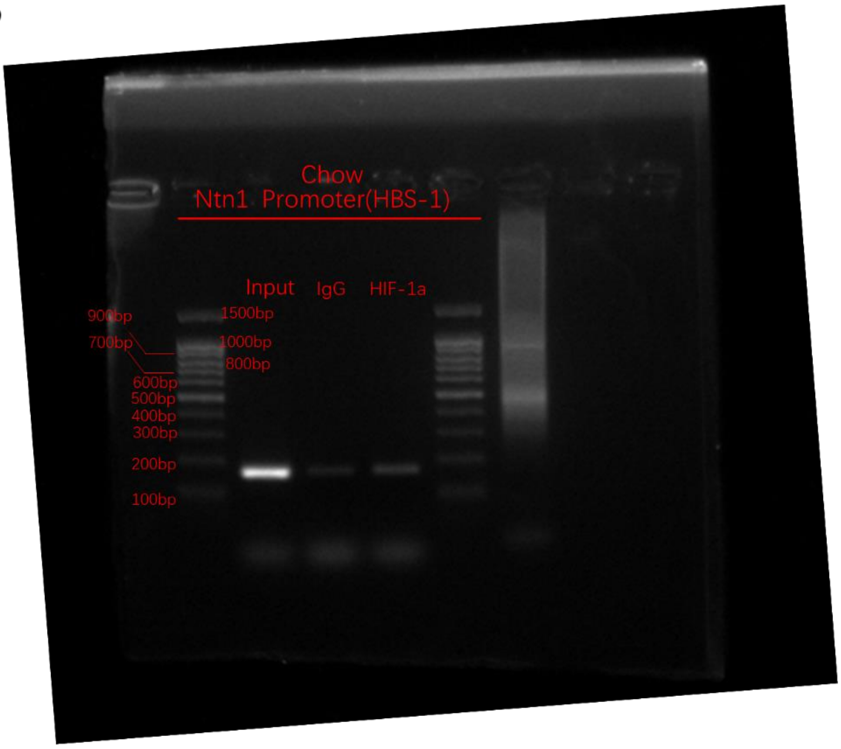

Supplementary Figure 9
Uncropped blots

Fig6 F

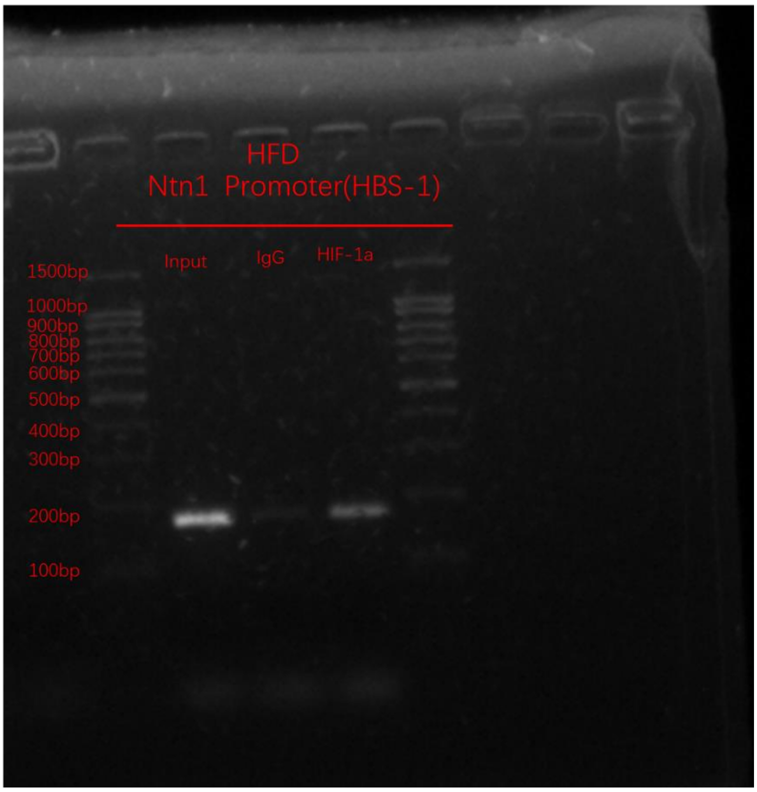

Supplement: Supplementary file 2 — Supplementary Information [file 42003_2026_9749_MOESM2_ESM.pdf]
