# Supplementary material for: Netrin-1 disrupt high-fat-diet-induced adipogenesis via the PPARγ and Wnt/β-catenin signaling pathways
Source: Commun Biol. 2026 Feb 21;9:471. doi: 10.1038/s42003-026-09749-x (PMC13035891; doi:10.1038/s42003-026-09749-x)
Supplement: Supplementary file 3 — Description of Additional Supplementary Files [file 42003_2026_9749_MOESM3_ESM.pdf]

Description of Additional Supplementary File

File name: Supplementary Data
Description: source data for graphical representations in the main figures are provided as Supplementary Data.
